# Improvement of triceps muscle weakness in patients with cervical spondylotic myelopathy using Korean medicine therapy including shinbaro-2 pharmacopuncture: A CARE-compliant case series

**DOI:** 10.1097/MD.0000000000043085

**Published:** 2025-07-04

**Authors:** Il-Hwan Ko, Yoon Jae Lee, In-Hyuk Ha, Ye-Seul Lee

**Affiliations:** a Jaseng Hospital of Korean Medicine, Seoul, Republic of Korea; b Jaseng Spine and Joint Research Institute, Jaseng Medical Foundation, Seoul, Republic of Korea.

**Keywords:** cervical spondylotic myelopathy, Korean medicine therapy, pharmacopuncture (shinbaro-2), triceps muscle weakness

## Abstract

**Rationale:**

Cervical spondylotic myelopathy (CSM) is the most prevalent spinal cord impairment among adults, primarily due to age-related degenerative changes. Although surgical interventions are commonly recommended for severe cases, the optimal timing and procedural complexities remain controversial. Evidence supporting the efficacy of Korean medicine (KM) as a treatment alternative for CSM is currently limited.

**Patient concerns:**

This report details 2 cases involving middle-aged Korean patients presenting with significant neck pain, upper extremity numbness, and muscle weakness. Case 1 involved a 50-year-old male (172 cm, 80 kg) experiencing left arm numbness, muscle weakness, and neck pain persisting for 3 months. Case 2 involved a 51-year-old male (182 cm, 85 kg) with persistent right arm and neck symptoms for 12 months.

**Diagnoses:**

Both patients were diagnosed with cervical compressive myelopathy associated with herniated intervertebral disc – Case 1 at cervical levels C4–C6 and Case 2 at cervical levels C6–C7.

**Interventions:**

Patients received integrated KM treatments, including acupuncture, pharmacopuncture (particularly Shinbaro pharmacopuncture), herbal medicine, and Chuna manual therapy.

**Outcomes:**

Both patients exhibited significant improvements in neck and arm pain, assessed via numeric rating scales. Additionally, manual muscle testing indicated notable enhancements in triceps brachii muscle strength.

**Lessons:**

The reported cases highlight KM treatment as a promising nonsurgical alternative for managing mild to moderate CSM, particularly in patients reluctant or unsuitable for surgery. KM treatments showed efficacy in improving pain, numbness, and muscular strength. Further rigorous studies are necessary to evaluate and confirm the broader applicability and effectiveness of KM approaches for CSM management.

## 1. Introduction

Cervical spondylotic myelopathy (CSM) is the most common type of spinal cord injury in adults^[1]^; it is a progressive disease characterized by degenerative changes affecting the vertebrae, intervertebral discs, facets, and associated ligaments.^[2]^ Age-related degeneration is the primary cause of CSM; however, spinal injuries to the discs due to traumatic events may aggravate the degenerative process in younger patients.^[3]^ In particular, due to its large range of motion, the cervical spine (C-SPINE) is highly susceptible to degenerative changes such as herniated intervertebral disc (HIVD), ligamentous hypertrophy, ossification, and osteophytosis (or osteophytic development). Degeneration of intervertebral discs and joints, including spondylosis, may result in the compression of the surrounding vascular and neural structures, which in turn affects disease severity.^[3]^ The cervical cord is subjected to compression due to disc herniation, ligamentum flavum hypertrophy, facet joint capsule thickening, and canal stenosis.^[4]^ Furthermore, as CSM can potentially lead to long-term disability and significant neurological impairments, identifying early symptoms for diagnosis and providing effective treatment before the progression to irreversible spinal cord damage is essential for maintaining patients’ quality of life.^[2]^

A higher prevalence of CSM has been reported in men than women, particularly among those engaged in labor-intensive occupations.^[5]^ Common initial symptoms of CSM include difficulties with hand dexterity, especially with finer tasks, as well as gait instability or falls.^[6]^ Upper extremity deficits are common in CSM, often due to lower motor neuron insults at the affected vertebral level, resulting in symptoms such as muscle weakness, muscle atrophy, myotonia, and hyporeflexia in the upper extremities.^[6]^ As for the management of CSM after monitoring disease progression, in cases of moderate or severe symptoms with persistent pain or muscle weakness, surgical treatments such as anterior cervical discectomy and fusion, laminectomy, and corpectomy^[7,8]^ are generally recommended. However, due to the complexity and difficulties of these procedures, and limited evidence on optimal timing concerning disease progression, further investigation is required.^[9]^ A recent study on the diagnostic criteria and grades of cervical canal (spinal) stenosis presented a classification system (Table 1).^[10]^ However, the epidemiology of CMS remains unclear due to diagnostic challenges, particularly in cases with degenerative causes.^[11]^

**Table 1:** Criteria for MRI-based diagnosis of CSM and grades.

MRI-based diagnosis	Grade
Loss of cerebrospinal fluid (CSF) 50% or more compared to the unaffected area, with no signs of cervical spine cord compression	Grade 1
Signs of cervical spine cord compression but no high-intensity signals in the spinal cord on T2-weighted MRI	Grade 2
Changes to high-intensity signals in the spinal cord	Grade 3

MRI = magnetic resonance imaging.

In Korean medicine (KM), various treatment modalities including acupuncture, herbal medicine, pharmacopuncture, and Chuna manual therapy, have been applied in clinical practice; however, few studies have investigated their effectiveness for CSM.

This study reports 2 cases of patients with CSM who presented with muscle weakness in the upper extremities and neck pain, and who experienced significant improvements in both symptoms following a combination of KM treatments. Both patients were diagnosed with compressive cervical myelopathy and complained of triceps muscle weakness in the upper arms. The patients voluntarily sought KM treatment at the hospital, resulting in symptom improvements. This study was approved by the Institutional Review Board of Jaseng Hospital of Korean Medicine (JASENG 2024-06-015). All participants were ambulatory, fully conscious, and competent to provide consent; therefore, each patient personally signed the informed consent form, and additional consent from legal guardians or next of kin was deemed unnecessary.

## 2. Case presentation

### 2.1. Patient information

#### 2.1.1. Case 1

The patient was a 50-year-old Korean male, measuring 172 cm in height and weighing 80 kg, with no significant family or medical history. He visited the hospital with complaints of numbness in the left upper arm, forearm, first, second, and fifth fingers, as well as upper arm muscle weakness and posterior neck pain, which had started 3 months before his initial visit during routine activities. He was diagnosed with compressive myelopathy at the cervical vertebrae C4, C5, and C6, and HIVD at C2, C3, C4, C5, C6, C7, and T1, based on C-SPINE magnetic resonance imaging (MRI) findings. Surgical treatment was recommended, but he declined surgery and returned to the hospital 3 months later. At his first visit, the Spurling test result was −/+; and the test results of Romberg, Hoffman, and Babinski signs were all negative.

#### 2.1.2. Case 2

The patient was a 51-year-old Korean male with anthropometric measurements of 182 cm and 85 kg and no significant family or medical history. He visited the hospital complaining of numbness in the right upper arm, forearm, and 1st and 2nd fingers, upper arm muscle weakness; and (posterior) neck pain while performing activities of daily living 12 months before the first visit. At the first visit, he underwent C-SPINE MRI, and was diagnosed with compressive myelopathy at the cervical vertebrae C6 and C7 and lumbar disc herniation at C3, C4, C5, C6, C7, T1, and T2, and started treatment at this hospital. At the time of his first visit, the Spurling test result was −/+, and the test results of Romberg, Hoffman, and Babinski signs were all negative.

### 2.2. Treatments

Both patients in this case report underwent KM treatment sessions, which included acupuncture, pharmacopuncture, herbal medicine, and Chuna manual therapy. For acupuncture, standardized disposable stainless steel filiform needles (0.25 mm × 30 mm, Dongbang Medical, Republic of Korea) were used, with bilateral Tianshu (ST25), Jianliao (TE14), Fengchi (GB20), and Fengfu (GV16) as t acupoints. The needle retention time was 15 minutes with electroacupuncture at 1 to 16 Hz AC. Pharmacopuncture was performed by injecting 2 to 6 cc of Shinbaro solution (Jaseng Herbal Medicine Dispensary), known for its anti-inflammatory and neurogenerative effects, into Ashi points. For herbal medicine therapy, Cheongpajeon-H (CPJH), Cheongpajeon-G (CPJG), Cheongpajeon-S (CPJS), Cheongwoongbaro-hwan, and Cheongpayanggeuntang were administered; the composition and doses are detailed in Tables 2 and 3). Chuna manual therapy included prone-lying C-SPINE adjustment, JS123, and upper-arm triceps relaxation/strengthening. Other KM treatments include indirect moxibustion, which effectively promotes blood circulation and pain relief through warming effects; cupping therapy, a method of removing blood states from the skin; and increasing circulation in the whole body and specific areas of the body.

**Table 2:** Details of the intervention (case 1).

Treatment type	Ingredients	Treatment details and frequency
Herbal medicine		
Cheongpajeon-H (CPJH)	*Eucommia ulmoides* 7.5 g *Acanthopanax sessiliflorus* 7.5 g *Achyranthes bidentata* 7.5 g *Saposhnikovia divaricata*, 7.5 g *Cibotium barometz* 7.5 g *Lycium chinense* 7.5 g, *Boschniakia rossica* 7.5 g *Cuscuta chinensis* 7.5 g Glycine max 7.5 g *Ostericum koreanum* 3.75 g *Atractylodes japonica* 3.75 g *Psoralea corylifolia* 3.75 g	30 min after each meal 2 times a day
Cheongpajeon-G (CPJG)	*Eucommia ulmoides* 7.5 g *Acanthopanax sessiliflorus* 7.5 g *Achyranthes bidentata* 7.5 g *Saposhnikovia divaricata*, 7.5 g *Cibotium barometz* 7.5 g *Lycium chinense* 7.5 g, *Boschniakia rossica* 7.5 g *Cuscuta chinensis* 7.5 g Glycine max 7.5 g *Ostericum koreanum* 3.75 g *Atractylodes japonica* 3.75 g *Psoralea corylifolia* 3.75 g *Drynaria fortune* 3.75 g *Phlomis umbrosa* 1.875 g	
Cheongpajeon-S (CPJS)	*Eucommia ulmoides* 7.5 g *Acanthopanax sessiliflorus* 7.5 g *Achyranthes bidentata* 7.5 g *Saposhnikovia divaricata*, 7.5 g *Cibotium barometz* 7.5 g *Lycium chinense* 7.5 g, *Boschniakia rossica* 7.5 g *Cuscuta chinensis* 7.5 g Glycine max 7.5 g *Ostericum koreanum* 3.75 g *Atractylodes japonica* 3.75 g *Psoralea corylifolia* 3.75 g *Drynaria fortune* 3.75 g *Phlomis umbrosa* 1.875 g *Drynaria fortune* 3.75 g *Asiasarum sieboldi* 3.75 g	
Cheongwoongbaro-hwan (CWBR) (pills)	*Acanthopanax sessiliflorus* 7.5 g *Cibot rhizome* 2 g *Eleutherococcus sessiliflorus* 7.5 g	
Pharmacopuncture		
Shinbaro (Jaseng Korean Medical Hospital, Namyangju, Republic of Korea)	*Cibotium barometz,* *Saposhnikovia divaricata,* *Eucommia ulmoides,* *Acanthopanax sessiliflorus,* *Ostericum koreanum,* *Angelica pubescens,* *Achyranthes japonica,* *Paeonia albiflora,* *Scolopendra subspinipes*	1 or 2 times a day Disposable syringe (Kovax-Syringe 2 mL, 26 G × 1 1/2)
Chuna manual therapy (CMT)		
Complex chuna manual therapy	Prone-lying high-velocity low-amplitude cervical manipulation JS 123 Post-isometric relaxation of Triceps brachii	Once a day

CSM = cervical spondylotic myelopathy.

**Table 3:** Details of the intervention (case 2).

Treatment type	Ingredients	Treatment details and frequency
Herbal medicine		
Cheongpajeon-G (CPJG)	*Eucommia ulmoides* 7.5 g *Acanthopanax sessiliflorus* 7.5 g *Achyranthes bidentata* 7.5 g *Saposhnikovia divaricata*, 7.5 g *Cibotium barometz* 7.5 g *Lycium chinense* 7.5 g, *Boschniakia rossica* 7.5 g *Cuscuta chinensis* 7.5 g Glycine max 7.5 g *Ostericum koreanum* 3.75 g *Atractylodes japonica* 3.75 g *Psoralea corylifolia* 3.75 g *Drynaria fortune* 3.75 g *Phlomis umbrosa* 1.875 g	
Cheongpajeon-S (CPJS)	*Eucommia ulmoides* 7.5 g *Acanthopanax sessiliflorus* 7.5 g *Achyranthes bidentata* 7.5 g *Saposhnikovia divaricata*, 7.5 g *Cibotium barometz* 7.5 g *Lycium chinense* 7.5 g, *Boschniakia rossica* 7.5 g *Cuscuta chinensis* 7.5 g Glycine max 7.5 g *Ostericum koreanum* 3.75 g *Atractylodes japonica* 3.75 g *Psoralea corylifolia* 3.75 g *Drynaria fortune* 3.75 g *Phlomis umbrosa* 1.875 g *Drynaria fortune* 3.75 g *Asiasarum sieboldi* 3.75 g	
Cheongwoongbaro-hwan (CWBR)(pills)	*Acanthopanax sessiliflorus* 7.5 g *Cibot rhizome* 2 g *Eleutherococcus sessiliflorus* 7.5 g	
Cheongpayanggeuntang (CPYG)	*Achyranthes bidentata* 7.5 g *Testudinis Plastrum* 2 g *Cibot rhizome* 2 g	
Pharmacopuncture		
Shinbaro (Jaseng Korean Medical Hospital, Namyangju, Republic of Korea)	*Cibotium barometz,* *Saposhnikovia divaricata,* *Eucommia ulmoides,* *Acanthopanax sessiliflorus,* *Ostericum koreanum,* *Angelica pubescens,* *Achyranthes japonica,* *Paeonia albiflora,* *Scolopendra subspinipes*	1 or 2 times a day Disposable syringe (Kovax-Syringe 2 mL, 26 G × 1 1/2)
Chuna manual therapy (CMT)		
Complex chuna manual therapy	Prone-lying high-velocity low-amplitude cervical manipulation JS 123 Post-isometric relaxation of Triceps brachii	Once a day

### 2.3. Outcome measures

Neck and arm pain were assessed using the Numeral Rating Scale (NRS), an 11-point scale using numbers for pain and paresthesia (numbness), 0 indicating no pain or paresthesia, and 10 indicating the most severe pain or paresthesia imaginable).

Triceps brachii muscle strength was evaluated using the manual muscle test (MMT), which classified muscle strength into 9 grades based on the patient’s strength against the resistance applied by the examiner and how the patient moved through the range of motion).

### 2.4. Follow-up and outcomes

**Figure 1.**
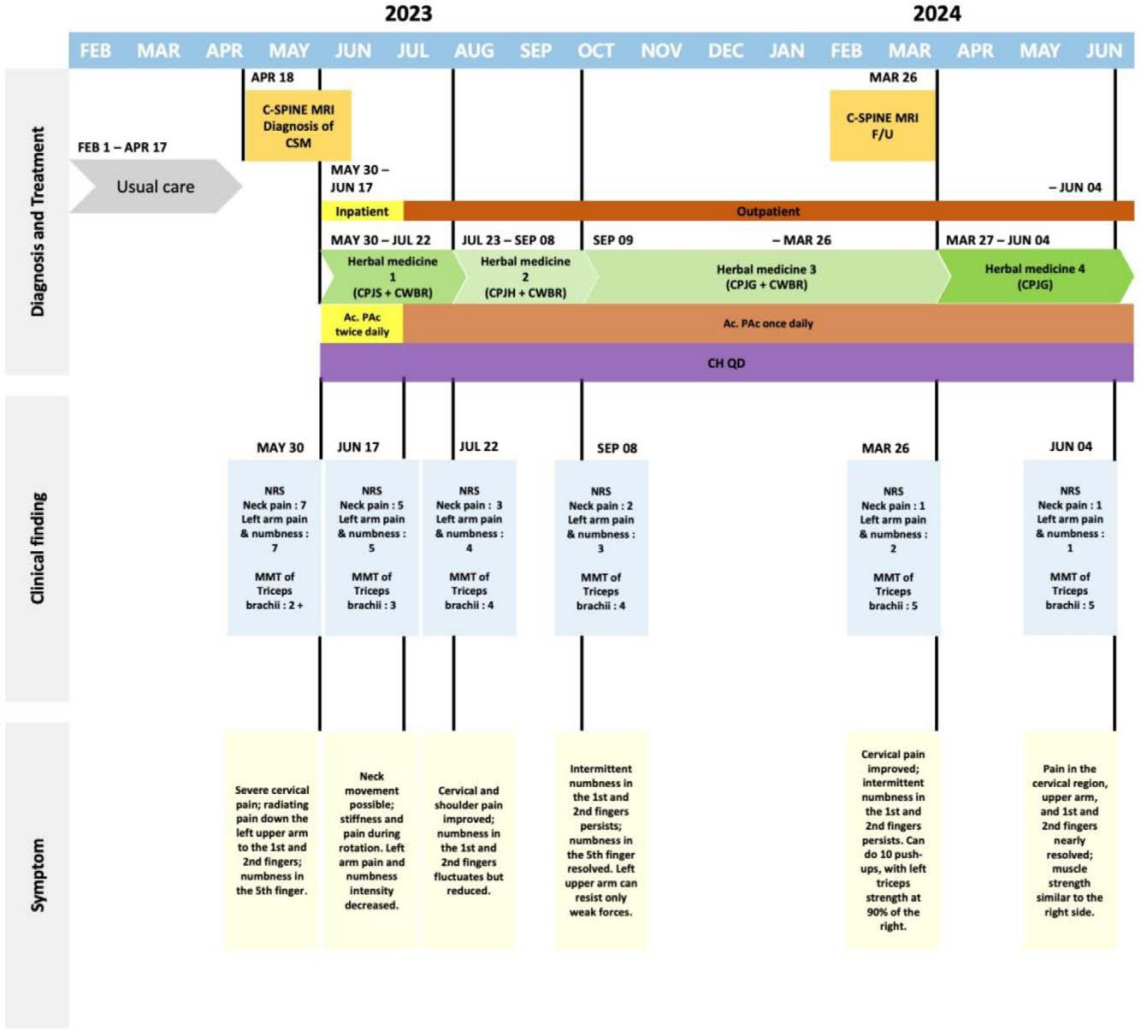
Summary of the treatment process with a timeline for Case 1.

**Figure 2.**
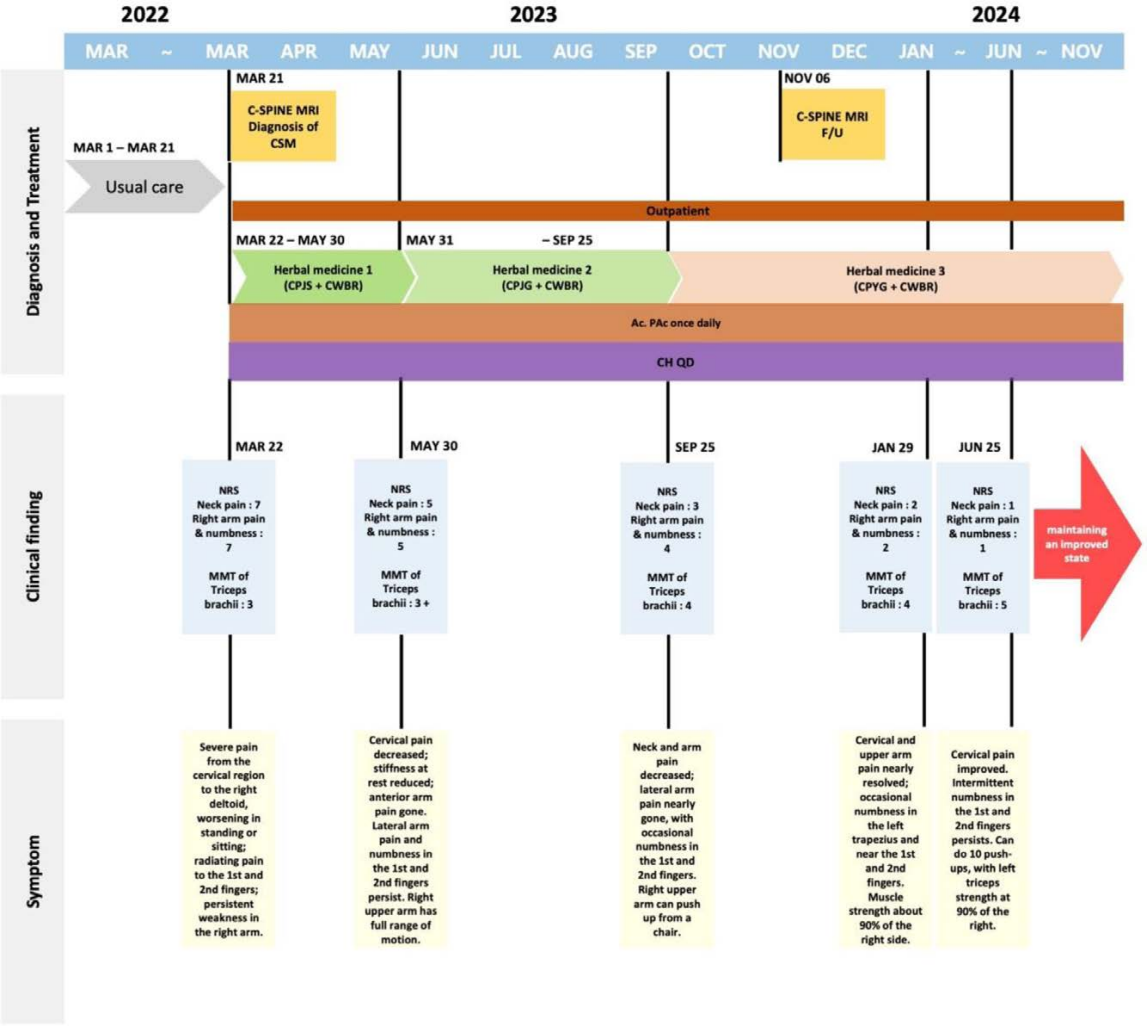
Summary of the treatment process with a timeline for Case 2.

**Figure 3.**
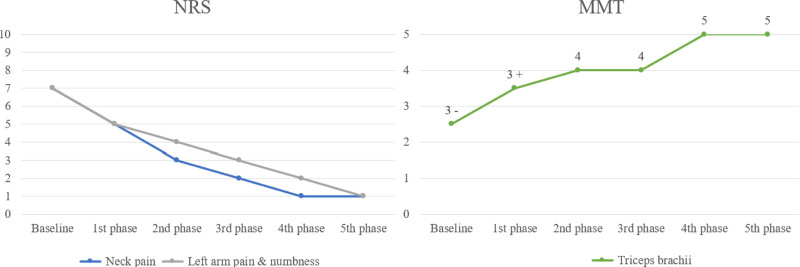
Case 1: Changes in NRS scores for neck pain, left arm pain, and numbness and MMT results for the triceps brachii. MMT = manual muscle testing, NRS = numeric rating scales.

**Figure 4.**
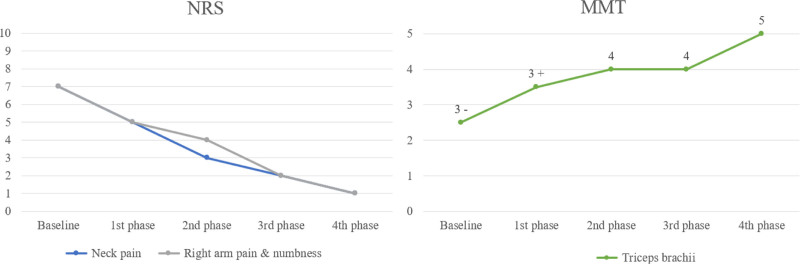
Case 2: Changes in NRS scores for neck pain, right arm pain, and numbness and MMT results for the triceps brachii. MMT = manual muscle testing, NRS = numeric rating scales.

The treatment processes for cases 1 and 2 are illustrated in Figures 1 and 2, with changes in outcomes, including NRS scores and MMT results, presented in Figures 3 and 4. Treatment phases were distinguished based on alterations in herbal prescriptions, adjusted according to symptom severity or notable symptomatic changes.

In detail, Case 1 exhibited significant clinical improvement, with left arm pain and numbness reducing from an initial NRS score of 7 to a final score of 1 by the fifth phase of treatment. Similarly, neck pain improved substantially from an initial NRS score of 5 to 1 by the fifth phase. Muscle strength assessment via MMT for the triceps brachii also showed remarkable enhancement, improving from a baseline rating of 3 to a rating of 5 in the fifth phase. Case 2 also demonstrated substantial improvement. Arm pain and numbness improved markedly from an initial NRS score of 7 to 1 by the fourth phase, and neck pain improved from an NRS score of 5 initially to 2 by the third phase. Likewise, triceps brachii muscle strength notably increased from a baseline MMT score of 3- to a rating of 5 by the fourth phase.

**Figure 5.**
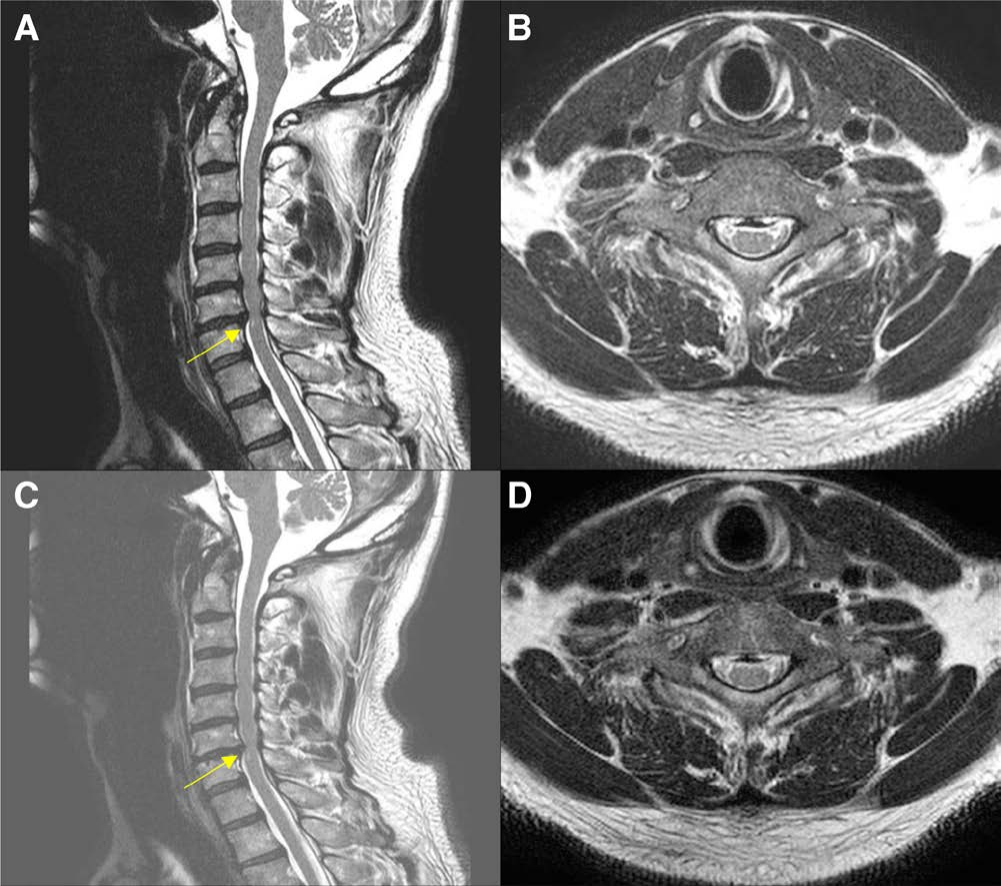
Case 1: (A) C-SPINE MRI. (B) Scanned on April 18, 2023. (C and D) Scanned on March 26, 2024. MRI = magnetic resonance imaging.

**Figure 6.**
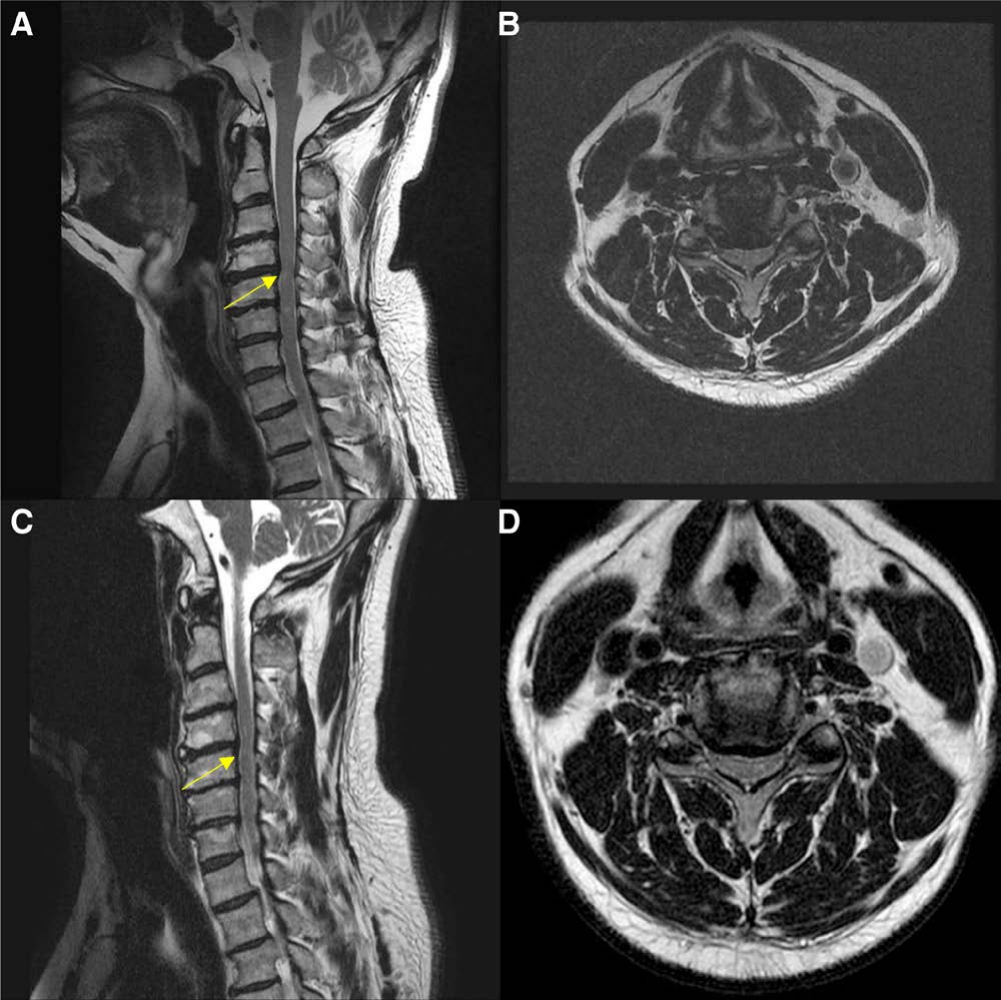
Case 2: (A) C-SPINE MRI. (B) Scanned on March 21, 2023. (C and D) Scanned on October 06, 2023. MRI = magnetic resonance imaging.

MRI images taken at baseline (i.e., at the initial visit or those brought by patients during their initial visit) and subsequent follow-up MRI images acquired during treatment are provided in Figures 5 and 6 for Cases 1 and 2, respectively. These results illustrate clear, quantifiable improvements in clinical symptoms and muscle strength following the integrated KM treatments administered in both cases.

## 3. Discussion

The 2 patients in this case report were diagnosed with CSM, presenting with muscle weakness in the upper arm triceps brachii, numbness in the forearm and the first and second fingers, and neck pain; their symptoms improved with KM treatment. CSM is a spinal cord disorder caused by spinal cord compression resulting from cervical canal stenosis due to degenerative changes in the C-SPINE. Upper motor neuron disorders, such as hyperreflexia and gait disturbance, are characteristic clinical features of CSM. It is generally observed that lower extremity symptoms, including gait disturbance, develop with the progression of CSM.^[12]^ However, the patients in this case report showed relatively mild symptoms with only upper extremity findings. Although the patients exhibited muscle weakness in the arms, both cases showed negative results for neurological signs such as Romberg, Hoffman, and Babinski signs; this is likely because these tests are more sensitive for detecting neurological signs in upper-level cervical vertebrae (C2–C4).^[13]^ The primary cause of cervical spinal stenosis leading to CSM includes degenerative changes in intervertebral discs and buckling of the ligamentum flavum. Cervical canal stenosis or compressive myelopathy, often detected by changes in spinal cord signal intensity, is commonly associated with CSM; however, the exact pathogenesis and epidemiology remain unclear due to diagnostic challenges.^[11]^

Neurological symptoms such as hypesthesia, motor impairments, and, in severe cases, loss of bowel and bladder control are considered red flags warranting emergency surgery. Surgical management is generally recommended for severe CSM, and previous studies indicate that 23 to 54% of patients who initially received nonsurgical treatment eventually undergo surgery as the disease progresses.^[14]^ Although surgery is recommended for severe CSM cases by neurosurgeons or orthopedic surgeons,^[8]^ the optimal timing of surgery is controversial due to the unpredicted disease course.^[9]^ Clinical guidelines recommend surgical intervention for moderate and severe cases or persistent deterioration of neurological symptoms. For patients with mild CSM, nonsurgical treatment, long-term monitoring, and structured rehabilitation programs are recommended; however, the quality of evidence is low, the strength of recommendations is weak, and detailed nonsurgical management methods are yet to be clearly established.^[8]^ Additionally, predicting long-term surgical outcomes for CSM is challenging, as recovery may take 6 to 12 months, and residual symptoms frequently persist. Older adults particularly face higher risks of complications after surgery, necessitating careful treatment decision-making.^[15]^ Currently, treatments beyond pain relief medications, including analgesics for neuropathic pain and antispastic drugs, remain limited.^[1]^

In the current debate on CSM treatment, a recent study reported that integrated KM treatment improved long-term pain, disability, and quality-of-life outcomes for patients with CSM.^[16]^ Therefore, this case report provides useful insights applicable to CSM treatment, particularly in patients with mild symptoms or those strongly averse to surgical intervention.

The herbal medicines administered included CPJH, CPJG, and CPJS, which were developed primarily to treat HIVD, spinal stenosis, and degenerative spinal lesions. *Harpagophytum procumbens*, a key ingredient, possesses anti-inflammatory, analgesic, and antioxidant effects.^[17]^ A recent study reported that in a murine model of lumbar spinal stenosis, CPJH effectively improved symptoms by maintaining the antioxidant defense mechanism by regulating iron metabolism.^[18]^ CPJS targets acute-phase patients with severe pain, incorporating *Asarum sieboldin*^[19]^ with anti-inflammatory effects and *Drynaria fortunei*^[20]^ to promote angiogenesis. CPJG is used in the recovery or later phases, containing *Phlomis umbrosa*^[21]^ and *Drynaria fortune*^[22]^ for bone growth and tissue regeneration. Shinbaro-2, the pharmacopuncture solution primarily used, has shown efficacy inflammation control and pain relief, enhancing motor function recovery by reducing neuroinflammation and supporting nerve regeneration.^[23]^ These mechanisms may underlie the improvements observed in patients’ upper extremity muscle strength and pain relief.

Chuna therapy, a manual therapy based on KM, was safely applied using the prone position C-SPINE adjustment method and JS123 technique, avoiding excessive force application contraindicated in CSM patients.^[24,25]^ Despite significant clinical improvement, there was no remarkable improvement in radiological findings on follow-up MRI examinations. Previous research indicated limited correlation between MRI findings of cervical cord compression and clinical symptoms, suggesting symptomatic improvements might not be mirrored radiologically.^[26]^

The limitations of this study include its case-report nature, small sample size, and lack of a control group, limiting generalizability. Additionally, the absence of standardized protocols for KM treatments complicates reproducibility. Despite these constraints, the findings highlight KM’s potential as an alternative treatment for mild CSM cases. Further large-scale, controlled clinical trials are necessary to confirm efficacy, elucidate exact mechanisms of action, and establish standardized treatment protocols.

## 4. Conclusion

In this case report, we present 2 patients with CSM complaining of triceps muscle weakness who achieved significant improvements in terms of NRS scores for neck pain, arm pain, and numbness, and the MMT result for the triceps brachii through the administration of integrated KM treatment. The results of this study indicate that KM treatment, whose effectiveness in the treatment of CSM has not been reported previously, may serve as a useful treatment option in clinical practice, especially in patients with difficulties in undergoing surgical treatment.

## Author contributions

**Conceptualization:** In-Hyuk Ha.

**Investigation:** Il-Hwan Ko.

**Methodology:** Yoon Jae Lee.

**Supervision:** Ye-Seul Lee.

**Writing – original draft:** Il-Hwan Ko.

**Writing – review & editing:** Yoon Jae Lee, In-Hyuk Ha, Ye-Seul Lee.

## References

[1] Davies BM Mowforth OD Smith EK Kotter MR. Degenerative cervical myelopathy. Bmj. 2018;360:k186. 29472200 10.1136/bmj.k186 PMC6074604

[2] McCormick JR Sama AJ Schiller NC Butler AJ Donnally CJ. Cervical spondylotic myelopathy: a guide to diagnosis and management. J Am Board Fam Med. 2020;33:303–13. 32179614 10.3122/jabfm.2020.02.190195

[3] Ferrara LA. The biomechanics of cervical spondylosis. Adv Orthop. 2012;2012:493605. 22400120 10.1155/2012/493605 PMC3287027

[4] Baptiste DC Fehlings MG. Pathophysiology of cervical myelopathy. Spine J. 2006;6(6 Suppl):190S–7S. 17097538 10.1016/j.spinee.2006.04.024

[5] Wu JC Ko C-C Yen Y-S. Epidemiology of cervical spondylotic myelopathy and its risk of causing spinal cord injury: a national cohort study. Neurosurg Focus. 2013;35:E10. 10.3171/2013.4.FOCUS13122 23815246

[6] Nandyala SV Jay Khanna A Hassanzadeh H. Pathophysiology, natural history, and clinical syndromes of cervical disc disease. In: Rothman-Simeone and Herkowitz’s the Spine. 7th ed. Elsevier, Inc, 2018:677–87.

[7] Bakhsheshian J Mehta VA Liu JC. Current diagnosis and management of cervical spondylotic myelopathy. Global Spine J. 2017;7:572–86. 28894688 10.1177/2192568217699208 PMC5582708

[8] Fehlings MG Tetreault LA Riew KD. A clinical practice guideline for the management of patients with degenerative cervical myelopathy: recommendations for patients with mild, moderate, and severe disease and nonmyelopathic patients with evidence of cord compression. Global Spine J. 2017;7(3 Suppl):70S–83S. 29164035 10.1177/2192568217701914 PMC5684840

[9] Tracy JA Bartleson JD. Cervical spondylotic myelopathy. Neurologist. 2010;16:176–87. 20445427 10.1097/NRL.0b013e3181da3a29

[10] Wei L Wei Y Tian Y Cao P Yuan W. Does three-grade classification of T2-weighted increased signal intensity reflect the severity of myelopathy and surgical outcomes in patients with cervical compressive myelopathy? A systematic review and meta-analysis. Neurosurg Rev. 2020;43:967–76. 31053986 10.1007/s10143-019-01106-3

[11] Nouri A Tetreault L Singh A Karadimas SK Fehlings MG. Degenerative cervical myelopathy: epidemiology, genetics, and pathogenesis. Spine (Phila Pa 1976). 2015;40:E675–93. 25839387 10.1097/BRS.0000000000000913

[12] Nurick S. The pathogenesis of the spinal cord disorder associated with cervical spondylosis. Brain. 1972;95:87–100. 5023093 10.1093/brain/95.1.87

[13] Paholpak P Jirarattanaphochai K Sae-Jung S Wittayapairoj K. Clinical correlation of cervical myelopathy and the hyperactive pectoralis reflex. J Spinal Disord Tech. 2013;26:E314–8. 23429310 10.1097/BSD.0b013e3182886edb

[14] Fehlings MG Tetreault LA Riew KD Middleton JW Wang JC. A clinical practice guideline for the management of degenerative cervical myelopathy: introduction, rationale, and scope. Global Spine J. 2017;7(3 Suppl):21S–7S. 10.1177/2192568217703088 PMC5684844 29164027

[15] Tetreault L Ibrahim A Côté P Singh A Fehlings MG. A systematic review of clinical and surgical predictors of complications following surgery for degenerative cervical myelopathy. J Neurosurg Spine. 2016;24:77–99. 26407090 10.3171/2015.3.SPINE14971

[16] Cho HW Park JH Yoo DH. Long-term follow-up of inpatients with cervical myelopathy who received integrative Korean medicine treatment: a retrospective analysis and questionnaire survey study. J Pain Res. 2022;15:801–12. 35370420 10.2147/JPR.S347410 PMC8974247

[17] Gxaba N Manganyi MC. The Fight against infection and pain: devil’s claw (Harpagophytum procumbens) a rich source of anti-inflammatory activity: 2011-2022. Molecules. 2022;27:3637. 35684573 10.3390/molecules27113637 PMC9182060

[18] Hong JY Kim H Lee J Jeon W-J Lee YJ Ha I-H. Harpagophytum procumbens inhibits iron overload-induced oxidative stress through activation of nrf2 signaling in a rat model of lumbar spinal stenosis. Oxid Med Cell Longev. 2022;2022:3472443. 36160714 10.1155/2022/3472443 PMC9492433

[19] Hashimoto K Yanagisawa T Okui Y Ikeya Y Maruno M Fujita T. Studies on anti-allergic components in the roots of Asiasarum sieboldi. Planta Med. 1994;60:124–7. 8202562 10.1055/s-2006-959432

[20] Huang ST Chang C-C Pang JS. Drynaria fortunei promoted angiogenesis associated with modified MMP-2/TIMP-2 balance and activation of VEGF Ligand/Receptors Expression. Front Pharmacol. 2018;9:979. 30298000 10.3389/fphar.2018.00979 PMC6160574

[21] Lee D Kim Y-S Song J. Effects of phlomis umbrosa root on longitudinal bone growth rate in adolescent female rats. Molecules. 2016;21:461. 27070559 10.3390/molecules21040461 PMC6273700

[22] Dong GC Ma T-Y Li C-H. A study of Drynaria fortunei in modulation of BMP–2 signalling by bone tissue engineering. Turk J Med Sci. 2020;50:1444–53. 32252500 10.3906/sag-2001-148 PMC7491309

[23] Hong JY Lee J Kim H. Shinbaro2 enhances axonal extension beyond the glial scar for functional recovery in rats with contusive spinal cord injury. Biomed Pharmacother. 2023;168:115710. 37862963 10.1016/j.biopha.2023.115710

[24] Ha IH Kim E-S Lee S-H. Cost-utility analysis of chuna manual therapy and usual care for chronic neck pain: a multicenter pragmatic randomized controlled trial. Front Med (Lausanne). 2022;9:896422. 35646995 10.3389/fmed.2022.896422 PMC9131099

[25] Lee J Cho J-H Kim K-W. Chuna manual therapy vs usual care for patients with nonspecific chronic neck pain: a randomized clinical trial. JAMA Netw Open. 2021;4:e2113757. 34259850 10.1001/jamanetworkopen.2021.13757 PMC8280970

[26] Kovalova I Kerkovsky M Kadanka Z. Prevalence and imaging characteristics of nonmyelopathic and myelopathic spondylotic cervical cord compression. Spine. 2016;41:1908–16. 27509189 10.1097/BRS.0000000000001842

